# Anti-psychotic drug prescription pattern for schizophrenia: Observation from a general hospital psychiatry unit

**DOI:** 10.4103/0019-5545.70996

**Published:** 2010

**Authors:** J.K. Trivedi, Mohan Dhyani, V.S. Yadav, S.B. Rai

**Affiliations:** Department of Psychiatry, C.S.M. Medical University (Formerly K. G. Medical University,) Lucknow - 226 003, U.P., India E-mail: jitendra.trivedi@gmail.com

Sir,

We did a study on the anti-psychotic drug prescription pattern for schizophrenia. The outpatients of the Department of Psychiatry, Chhatrapati Shahuji Maharaj Medical University, U.P., Lucknow (CSMMU, Lucknow) were taken into the study. The first five patients, who were diagnosed as suffering from schizophrenia, as per the diagnostic criteria of the International Classification of Disease, Tenth revision (ICD-10),[1] were included in the study from 20 consecutive OPDs, thus making a sample of 100 patients.

The sample consisted mostly of male patients. This is in concordance with the trends observed at our center and could be a reflection of the cultural norms.

The sample comprised of mostly young patients. Schizophrenia is an illness starting early and our sample reflects that.

It was observed that the most frequently prescribed total daily dose of anti-psychotic equivalent to Chlorpromazine was between 101–400 mg/day. This was in accordance with the findings world over with SGAs becoming the first line of treatment.

A significant group of patients, however, were prescribed a combination of first generation anti-psychotics (FGAs) and second generation anti psychotics (SGAs). Poly-pharmacy therefore was common. The use of poly-pharmacy can be explained by a number of reasons. First, when a patient is doing poorly a physician may add a medication to what is currently prescribed; when the patient shows some improvement the physician is reluctant to change this regime.[2] Second, when changing medications, a physician noting an improvement while a new medication is being decreased may stop the cross-titration and continue coprescribing both anti-psychotic medications.[2,3] Third, shorter hospital stays may increase pressure for poly-pharmacy.[2] Fourth, a recent trend is the pharmaceutical companies promoting the use of their anti-psychotics in combination with other medications.[2] Fifth, clinicians may feel that different medications are better for different symptoms, even though the drugs are similar.[2] Finally, busy doctors may be more inclined to prescribe multiple drug prescriptions than doctors who have more time and are under less pressure.[4] Whatever the reason, the fact that poly-pharmacy has been shown across several studies to be a relatively common practice suggests a need for research to determine whether the approach to treatment with two anti-psychotic medications is warranted, and if so, under what circumstances.[5]

The findings from the study include some expected findings with SGAs being the primary choice of treatment. There are, however, some interesting findings, such as polypharmacy with more than one anti-psychotic. Hopefully future studies will throw more light on the prescription patterns in this region.
